# Transcriptional Responses Reveal Similarities Between Preclinical Rat Liver Testing Systems

**DOI:** 10.3389/fgene.2018.00074

**Published:** 2018-03-20

**Authors:** Zhichao Liu, Brian Delavan, Ruth Roberts, Weida Tong

**Affiliations:** ^1^Division of Bioinformatics and Biostatistics, National Center for Toxicological Research, U.S. Food and Drug Administration, Jefferson, AR, United States; ^2^Department of Biosciences, University of Arkansas at Little Rock, Little Rock, AR, United States; ^3^ApconiX, Alderley Edge, United Kingdom; ^4^Department of Biosciences, University of Birmingham, Birmingham, United Kingdom

**Keywords:** toxicogenomics, preclinical models, liver, gene expression, bioinformatics

## Abstract

Toxicogenomics (TGx) is an important tool to gain an enhanced understanding of toxicity at the molecular level. Previously, we developed a pair ranking (PRank) method to assess *in vitro* to *in vivo* extrapolation (IVIVE) using toxicogenomic datasets from the Open Toxicogenomics Project-Genomics Assisted Toxicity Evaluation System (TG-GATEs) database. With this method, we investiagted three important questions that were not addressed in our previous study: (1) is a 1-day *in vivo* short-term assay able to replace the 28-day standard and expensive toxicological assay? (2) are some biological processes more conservative across different preclinical testing systems than others? and (3) do these preclinical testing systems have the similar resolution in differentiating drugs by their therapeutic uses? For question 1, a high similarity was noted (PRank score = 0.90), indicating the potential utility of shorter term *in vivo* studies to predict outcome in longer term and more expensive *in vivo* model systems. There was a moderate similarity between rat primary hepatocytes and *in vivo* repeat-dose studies (PRank score = 0.71) but a low similarity (PRank score = 0.56) between rat primary hepatocytes and *in vivo* single dose studies. To address question 2, we limited the analysis to gene sets relevant to specific toxicogenomic pathways and we found that pathways such as lipid metabolism were consistently over-represented in all three assay systems. For question 3, all three preclinical assay systems could distinguish compounds from different therapeutic categories. This suggests that any noted differences in assay systems was biological process-dependent and furthermore that all three systems have utility in assessing drug responses within a certain drug class. In conclusion, this comparison of three commonly used rat TGx systems provides useful information in utility and application of TGx assays.

## Introduction

Toxicogenomics (TGx) combines toxicology with genomics or other high throughput molecular profiling technologies, offering a powerful method to study the underlying molecular mechanisms of toxicity (Nuwaysir et al., 1999; Aardema and MacGregor, 2002). Since, it was first described some 18 years ago (Nuwaysir et al., 1999), TGx has played an important role in various aspects of toxicology including mechanistic studies, predictive toxicology and the development of safety biomarkers (Chen et al., 2012).

Toxicogenomic approaches can be broadly categorized into three purposes: predictive toxicology, risk assessment, and mechanistic studies (Suter et al., 2004; Chen et al., 2012). For example, Fielden et al. (2007) developed a short-term (5 day) repeated dose TGx assay in rat to predict non-genotoxic hepatocarcinogenicity with a sensitivity and specificity of 86 and 81%, respectively. Other studies have addressed various questions of applying TGx including optimal treatment duration and sample size for a better predictive performance (Liu et al., 2011; Gusenleitner et al., 2014; Matsumoto et al., 2015; Liu S. et al., 2017). TGx has also been used in semi-quantitative risk assessment such as defining points of departure and benchmark dosing (Yang et al., 2007; AbdulHameed et al., 2016; Chauhan et al., 2016; Dean et al., 2017; Farmahin et al., 2017; Kawamoto et al., 2017). Most widely used application of TGx approaches is to understand the molecular mechanisms of different toxicological endpoints (Ellinger-Ziegelbauer et al., 2008; Blomme et al., 2009; Rodrigues et al., 2016; Hendrickx et al., 2017; Rueda-Zárate et al., 2017). More recently, in addition to gene expression profiling, the study of microRNAs (Wang et al., 2009; Yang et al., 2012; Ward et al., 2014; Liu et al., 2016) and long non-coding RNAs (lncRNAs) (Aigner et al., 2016; Dempsey and Cui, 2017) are emerging as new technologies to be integrated into this field powered by next-generation sequencing technologies (Yu et al., 2014).

In drug development, TGx has been added as an endpoint to existing preclinical study designs to gain more information from these studies. For example, in studies of liver toxicity, preclinical assessment in rodents may use primary rat hepatocytes or may use single dose *in vivo* studies (24 h) or repeat dosing up to 28-days. Each of these test systems may serve a different purpose; *in vitro* studies using primary rat hepatocytes may be used for mechanistic and/or cytotoxicity assessments whereas single and repeat dose toxicity studies are used to determine tolerability and target organ toxicity. The addition of TGx to each of these study types has generated additional data of use in assessment of toxicological risk and mechanisms. Other researchers have compared different testing systems for analysis of such endpoints as identification of biomarkers (Kondo et al., 2009) and gene expression-induced by genotoxic carcinogens (Watanabe et al., 2007). However, a systematic comparison of the value of TGx data generated in the different test systems has not been fully assessed.

Unlike decades ago, there are now several large publicly available toxicogenomic datasets such as the Open Toxicogenomics Project-Genomics Assisted Toxicity Evaluation System (TG-GATEs) database (Uehara et al., 2010; Igarashi et al., 2015), DrugMatrix (Ganter et al., 2005) and PredTox (Suter et al., 2011), providing tremendous opportunities for comparing preclinical testing systems. For example, open TG-GATEs used four standard preclinical study designs to generate TGx data (Ippolito et al., 2015; Bell et al., 2016; Liu et al., 2016; Sutherland et al., 2016). Using TG-GATEs data, we developed a ‘topic modeling’ approach to explore the underlying relationships between different TGx assay systems (Lee et al., 2014, 2016) and other toxicological assessments such as high throughput screening assay data from the Tox21 project (Lee et al., 2016).

In our previous study, we developed a Pair Ranking (PRank) method to assess the potential of *in vitro* to *in vivo* extrapolation (IVIVE) among three TGx assay systems (two *in vitro* assays using rat or human hepatocytes and a 28-day repeat-dose rat model) (Liu Z. et al., 2017). The study had an emphasis on assessing the IVIVE potential for different endpoints of drug-induced liver injury (DILI). It was concluded that the *in vitro* assay using primary rat hepatocytes and rat *in vivo* 28-day repeated dose models had high IVIVE potential for most DILI endpoints. However, several important questions remain for prediction of liver responses. Firstly, will a short-term *in vivo* assay (1-day experiment to detect acute response) correlate with a standard long-term *in vivo* repeated dose study (28-day study)? Secondly, are differences and similarities dependent upon biolgocial processes? Finally, can the different TGx assay systems distinguish compounds from different therapeutic categories?

In this study, we analyzed preclinical rat test system data from TG-GATEs comprising 131 compounds in three assays – (1) an *in vitro* study with rat primary hepatocytes (denoted as InVitro hereafter), (2) a rat *in vivo* single-dose treatment wih sample collection after 24 h (denoted as InVivo_S), and (3) a rat 28 day repeat-dose study (denoted as InVivo_R hereafter). Comparative analysis among these three assay systems were analyzed using PRank. Additonal useful comparisons were genererated by limiting the analyses firstly to compounds from certain therapeutic categories and secondly to gene sets representing specific toxicogenomic pathways.

## Materials and Methods

### Toxicogenomics Datasets

The open TG-GATEs^1^ was employed to investigate preclinical TGx assay systems in rats (Igarashi et al., 2015). Three rat toxicogenomic data sets from the TG-GATEs Phase I study were included covering 131 compounds from different therapeutic categories. The rat *in vitro* data had three concentrations (low, medium, and high) and three treatment durations (2, 4, and 24 h). The rat *in vivo* single dose also used three doses (low, medium, and high) and the samples were collected at four different timepoints after treatment (3, 6, 9, and 24 h). The *in vivo* repeated dose data was generated under the standard *in vivo* experiment design with three doses (low, medium, and high) and four treatment durations (3, 7, 14, and 28 days), where the rat liver tissue was isolated 24 h after treatment. In this study, we focused on the highest concentration/dose and longest treatment duration of 120 common compounds among the three assay systems for each assay system (the data used are available from **Supplementary Table S1**). Specifically, (1) “InVitro” is the data from *in vitro* assay with rat primary hepatocytes treated with the highest dose and the sample is collected 24 h after treatment, (2) “InVivo_S” is the data from rat *in vivo* single high dose and the sample is collected 24 h after treatment, and (3) “InVivo_R” is repeated dose daily with highest dose for 28 days. More details on concentration and dose definition are listed in our previous study (Liu Z. et al., 2017) and elsewhere (Igarashi et al., 2015).

### Microarray Data Normalization and Differentially Expressed Genes (DEGs) Calculation

The microarray data from three rat TGx systems was processing using Factor Analysis for Robust Microarray Summarization (FARMS) (Hochreiter et al., 2006) with a custom CDF file from BRIANARRAY^2^. The details were as described previously (Hochreiter et al., 2006; Liu Z. et al., 2017). Replicate measurements were collapsed to one measurement per gene. The collapsed data can be downloaded from http://dokuwiki.bioinf.jku.at/doku.php/tgp_prepro. The downloaded data were transformed as MAT File Format as an input for further analysis. For each compound in each assay system, the fold change values were generated by comparing the treatment group vs. matched control group for each time and concentration/dose condition.

### Therapeutic Categories

The Anatomical Therapeutic Chemical (ATC) classification system was used to group the compounds into different therapeutic classes. The ATC classification system has five levels of code to characterize a chemical/drug based on (1) the system/organ it acts on, (2) its therapeutic use, (3) its pharmacological functions, (4) its chemical properties, and (5) the chemical itself. In this study, the second-level of ATC codes indicating the main therapeutic group were used (see **Supplementary Table S1**).

### Toxicity Pathways Related Gene Sets

The gene sets related to different toxicity pathways were extracted from the Comparative Toxicogenomics Database (CTD) (Davis et al., 2017), which aims to illustrate how environmental chemicals affect human health. Specifically, the gene and pathway relationship data were downloaded from http://ctdbase.org/downloads/. There are a total of 135,815 gene and pathway relationships. Due to the gene symbols (Entrez Gene IDs) in CTD database was based on homo sapiens, we mapped Entrez gene IDs from homo sapiens to Rattus norvegicus based on NCBI HomoloGene build 68^3^. We clustered the genes based on their related pathways and kept the pathways containing more than 200 genes for further analysis (see **Supplementary Table S2**).

### Pair Ranking Method (PRank)

The Pair Ranking (PRank) method was used to compare the three rat TGx assay systems (Liu Z. et al., 2017). First, the pairwise compound similarity of any two compounds within an assay system was calculated using their biologically significant genes which were the top and down 200 ranked genes by their fold change values. The total number of 400 genes as the compound signatures were used for similarity calculation. The Dice’s coefficient was employed to measure the similarity between the transcriptional profiles of compounds, which were suggested by SEQC I study (Wang et al., 2014). In this study, the overlapped genes were counted by taking into consideration of their regulation direction and the Dice’s coefficient were calculated by using the following equation,

(1) $$\mathrm{Dice’s coefficient}=\frac{2(N_{i,j,\mathrm{up}}+N_{i,j,\mathrm{down}})}{400+400}=\frac{N_{i,j,\mathrm{up}}+N_{i,j,\mathrm{down}}}{400}$$

(2) $$\mathrm{stability ratio}=\frac{\mathrm{mean}(\sum_{i=1}^{n}\mathrm{Dice}\_\mathrm{inter})}{\mathrm{mean}(\sum_{i=1}^{n}\mathrm{Dice}\_\mathrm{across})}$$

where, *N*_*i*,*j*,up_ and *N*_i,_*_j,down_* denote the number of overlapped the up/down regulated genes between compound *i* and compound *j*, respectively. Then, each pair was ranked from high to low by the pairwise similarity. Lastly, the PRank score was calculated between any two assay systems by using receiver operating characteristic (ROC) curve and the area under the curve (AUC). For ROC-AUC analysis, we need to transform the ranked Dice’s coefficient to binary values (positive and negative: 0/1). In this study, the Dice’s coefficient value more than 0.4 was selected as cut-off, which is close to 95% quantile. The ROC-AUC analysis was conducted by using function *perfcurve.m* from MATLAB R2016a.

To investigate whether the compounds within a therapeutic category were more similar than across therapeutic categories, we used the following formula,

equation

where, *n* is the number of compound pairs. For inter therapeutic category, the compound pairwise similarity was generated by calculating the Dice’s coefficients between any two compounds from the same category. For across therapeutic categories, the pairwise similarity was generated between compounds from the different therapeutic categories. Finally, we calculated the stability ratio between inter therapeutic and across therapeutic categories to investigate whether the assay system could distinguish one therapeutic category to another. If the stability is more than 1, it means that the similarity among compounds for inter therapeutic category is more than across therapeutic category, indicating the similarity based on toxicogenomic profiles is capable of distinguishing the compounds from one therapeutic category to another.

For compound pairwise similarity calculations using the gene sets from different toxicogenomic pathways, we followed the following procedures. First, we mapped each gene set derived from toxicogenomic pathways to rat genes represented by the microarrays used in open TG-GATEs. Then, we retained the overlapped genes with fold change more than 1.5 for each compound as individual signatures. Finally, we calculated Dice coefficients between any two compounds based on the generated signatures in each system.

### Percentage of Overlapping Pathways (POP)

The concordance among the three assay systems were also assessed in the different KEGG pathways. Specifically, the 400 genes for each compound in each assay system was input to the Database for Annotation, Visualization and Integrated Discovery (DAVID) software to carry out KEGG pathway analysis (Huang et al., 2008). The pathways with a Benjamini–Hochberg adjusted *p*-value less than 0.05 were considered as statistically significant pathways. Then, the enriched KEGG pathways in each assay systems were ranked based on frequency of pathways perturbed by the compounds (*p* ≤ 0.05). Finally, the POP represented the number of common pathways between any two assay systems divided by L, the number of pathways in each of subset of ranked pathway list. In this study, L was set from 5 to 60.

#### Chemical Structure Similarity

The chemical structure of 120 common compounds could be found from our previous study (Liu Z. et al., 2017). The Pipeline Pilot 8.0 (Accelrys, Biovia, and Dassault Systems) was used to calculate the compound pairwise similarity based on their functional class fingerprints (FCFPs) with a radius of FCFP-4. The compound pairwise chemical similarities were listed in **Supplementary Table S1**.

##### Code availability

The scripts and processed data in this study were available in https://github.com/iguana128/Frontier-source-codes.

## Results

### Detection Power of Three TGx Assay Systems

**FIGURE 1:**
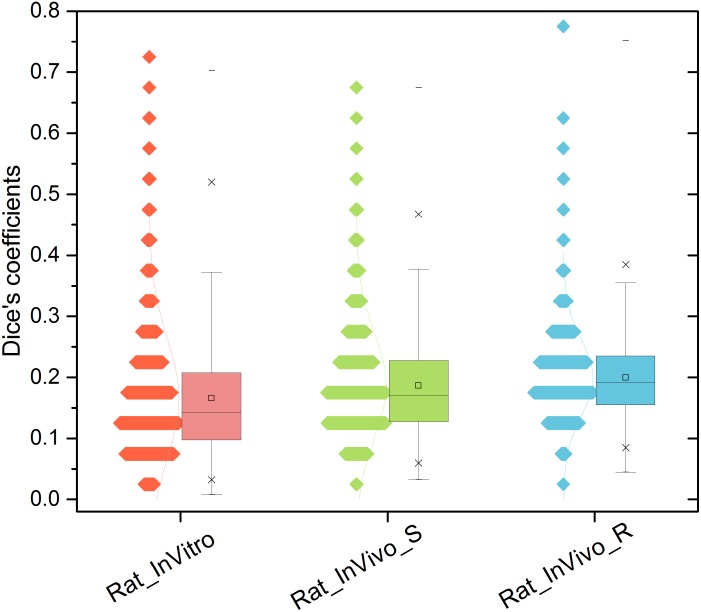
Distribution of compound pairwise similarity in the gene level across the three rat toxicogenomics assay systems: Dice’s coefficient was calculated based on top and down 200 genes ranked by fold changes for any two compounds in each system.

We first examined each assay’s ability to differentiate drug pairs. **Figure 1** illustrates the pairwise similarity distribution for the three TGx assay systems. The average Dice’s coefficients in the three assay systems were ranked as InVivo_R (Dice’s coefficient = 0.200) > InVivo_S (Dice’s coefficient = 0.187) > InVitro (Dice’s coefficient = 0.166) (see **Supplementary Table S3**). The low Dice’s coefficients indicated that all three TGx assay systems could differentiate one compound pair to another, where the InVivo_R assay seems to be less sensitive compared to other two assays.

Read-across have been widely applied to risk assessment based on chemical structure similarity (Vink et al., 2010; Rand-Weaver et al., 2013). Recently, the read-across concept has been expanded to integrate biological data profiles such as TGx and cell-based *in vitro* assays (Zhu et al., 2016). Here, the drug pairs in each assay system were compared with the compound pairwise chemical similarity (Dice coefficients > 0.2). It was illustrated that the correlation between assay systems and chemical space was low with the Pearson’s correlation coefficients of 0.30, 0.20, and 0.21 for chemical space vs. InVitro, InVivo_S, and InVivo_R, respectively (**Supplementary Figure S1**). The difference between the chemical space and toxicogenomic space suggested that the read-cross can be improved by combining the information from both chemistry and toxicogenomics spaces.

### Therapeutic Class Analysis

**FIGURE 2:**
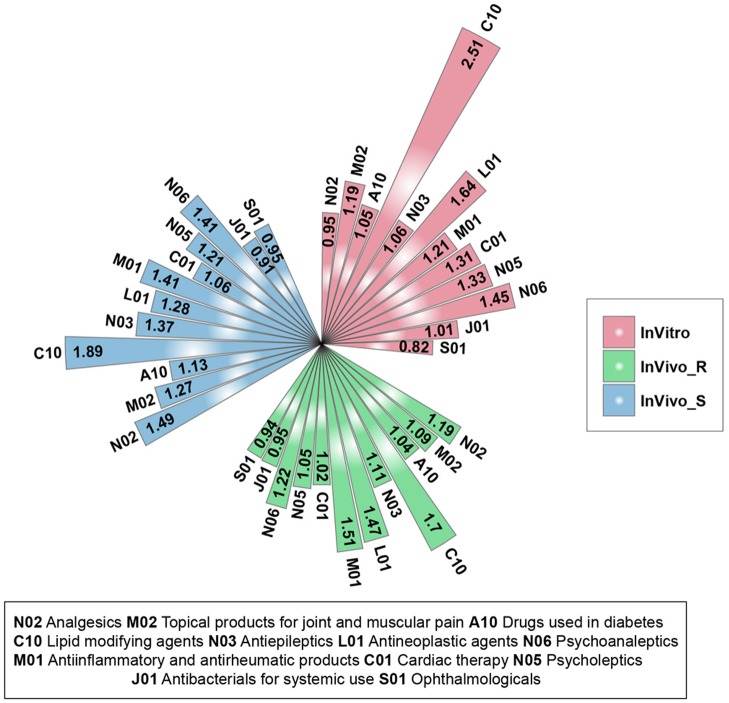
Stability ratios for the 12 therapeutic categories in each assay system: for each assay system, the stability ratio was calculated based on the average of Dice’s coefficient of inter and across therapeutic categories.

We further investigated whether the three TGx assay system could be utilized to discriminate different therapeutic categories. There was a total of 12 therapeutic categories with at least five compounds (***N02****-Analgesics;* ***M02****-Topical products for joint and muscular pain;* ***A10****-Drugs used in diabetes;* ***C10****-Lipid modifying agents;* ***N03****-Antiepileptics;* ***L01****-Antineoplastic agents;* ***M01****-Antiinflammatory and antirheumatic products;* ***C01****-Cardiac therapy;* ***N05****-Psycholeptics;* ***N06****-Psychoanaleptics;* **J01**-Antibacterials for systemic use; **S01**-Ophthalmological***s***) (**Supplementary Figure S2**). For each therapeutic category and each assay system, the stability ratios were calculated by comparing the mean value between and across categories. Almost all the therapeutic categories in each assay system had a stability ratio of more than 1 (**Figure 2**), suggested that the assay systems could distinguish the different therapeutic categories from each other. Among 12 therapeutic categories, the high stability ratios of ***C10****-Lipid modifying agents* was observed in all three assay systems, indicating the lipid modifying agents including clofibrate, fenofibrate, gemfibrozil, nicotinic acid, simvastatin could be distinguished from compounds in other therapeutic categories in TGx assay systems. Furthermore, **J01**-Antibacterials for systemic use and **S01**-Ophthalmological***s*** with stability ratios less than 1 in all three assay systems, showing the lower discrimination power of TGx assay systems for compounds from these two therapeutic categories. It could be seen that the high proportion of compounds were overlapped between the some therapeutic categories (**J01**-Antibacterials for systemic use and **S01**-Ophthalmologicals, and **M01**-Antiinflammatory and antirheumatic products and **M02**-Topical products for joint and muscular pain) due to the multiple therapeutic uses of these compounds, indicating the complexity of off-target space of these compounds, which may partially explain the unsatisfactory discrimination power.

### Concordance Among Three TGx Assay Systems

**FIGURE 3:**
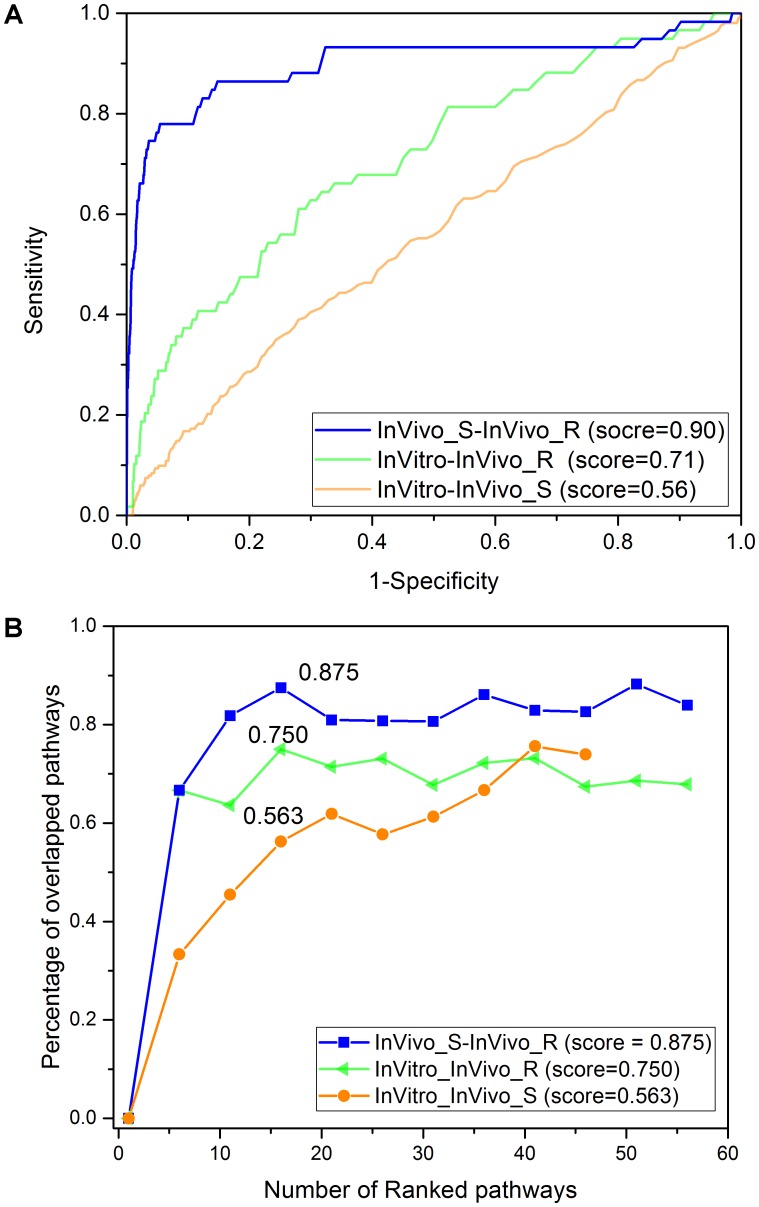
The concordance among the three rat toxicogenomics assay systems: **(A)** PRank methodology based on the top and down 200 genes based on fold change values. **(B)** The percentage of overlapping KEGG pathways based on over-represented KEGG pathways using Fisher’s exact test with adjusted *p*-value less than 0.05.

**Figure 3A** shows the concordance among three assay systems (InVitro, InVivo_S, and InVivo_R) based on the PRank scores. The highest concordance was noted for the InVivo_S (24 h) and InVivo_R (28-day) with a PRank score 0.90, suggesting the potential to replace long-term treatments with a 1-day experiment using a single dose treatment without loss of prediction. As reported in our previous study, the InVitro and InVivo_R also had a relatively high PRank score (0.71), suggesting a good IVIVE potential (Liu Z. et al., 2017). However, the concordance between InVitro and InVivo_S (PRank score = 0.56) was lower despite the same treatment duration in these two assay systems.

The concordance among the three assay systems was compared at the pathway level. Specifically, the percentage of overlapped pathways (POP) was calculated based on shared over-represented KEGG pathways (Fisher’s exact test with adjusted *p*-value < 0.05) between any two assay systems. As illustrated in **Figure 3B**, the highest concordance was for the two *in vivo* systems (POP value = 0.875), followed by InVitro-InVivo_R (POP value = 0.750) and InVitro-InVivo_S (POP value = 0.563). Therefore, a similar pattern was found at both the gene and pathway level. Furthermore, pathways related to lipid metabolism such as s*teroid hormone biosynthesis* and *fatty acid metabolism* were consistently over-represented in the three assay systems (**Table 1**).

**Table 1:** The overlapping KEGG pathways among the three assay systems.

KEGG entry	Pathways names	Categories
rno00140	Steroid hormone biosynthesis	Lipid metabolism
rno00071	Fatty acid metabolism	Lipid metabolism
rno00330	Arginine and proline metabolism	Amino acid metabolism
rno00280	Valine, leucine, and isoleucine degradation	Amino acid metabolism
rno00480	Glutathione metabolism	Metabolism of other amino acids
rno00982	Drug metabolism	Xenobiotics biodegradation and metabolism
rno00980	Metabolism of xenobiotics by cytochrome P450	Xenobiotics biodegradation and metabolism
rno00830	Retinol metabolism	Metabolism of cofactors and vitamins
rno03320	PPAR signaling pathway	Endocrine system

### Toxicity Pathway Analysis

**FIGURE 4:**
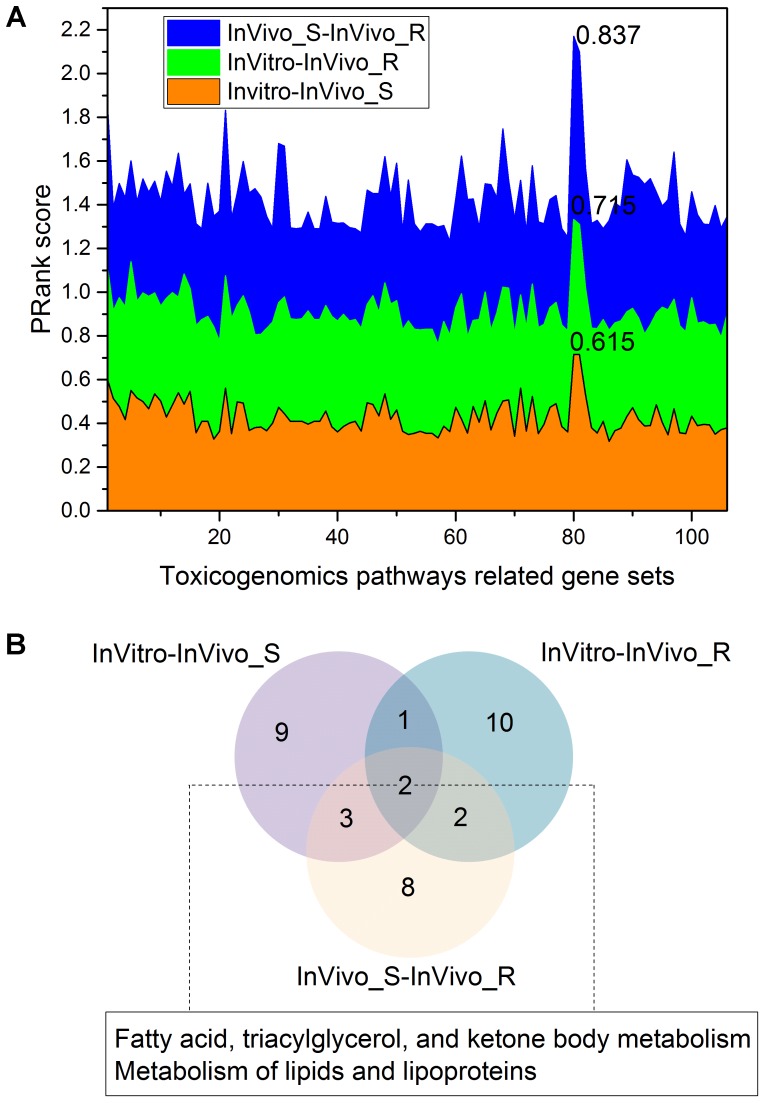
The concordance among the three rat toxicogenomics assay systems for gene sets related to different toxicogenomic pathways: **(A)** the stacked plots of PRank scores for different gene sets in the three assay systems; **(B)** a Venn diagram of the top 15 gene set-related toxicogenomic pathways based on the PRank score ranking in each rat assay systems.

We further investigated the concordance among the three assay systems when limiting the genes to specific toxicity pathways. The >135K gene-pathway relationships in CTD were employed, and a total of 106 toxicity pathways related genes sets with at least 200 genes for each were extracted (see **Supplementary Table S2**). **Figure 4A** depicts the concordance among the three assay systems in different toxicity pathway. The concordance among the three assay systems in the gene sets level was consistent with the finding in the whole gene/pathway level with a concordance ranking as InVivo_S-InVivo_R > InVitro-InVivo_R > InVitro-InVivo_S. We furthermore compared the top 15 common gene sets related pathways in the three comparisons based on the PRank scores (**Figure 4B**). We found that two lipid related pathways, i.e., *Metabolism of lipids and lipoproteins* and *Fatty acid, triacylglycerol, and ketone body metabolism* were common in all three comparisons, which is also consistent with the finding in the whole gene/pathway level. Furthermore, the similar conclusion was also drawn based on the stability analysis, which suggested the *lipid modifying agents* (C10) were highly discriminated in all three TGx testing systems.

#### Confirmation Based on Multiple Time and Dose Points

**FIGURE 5:**
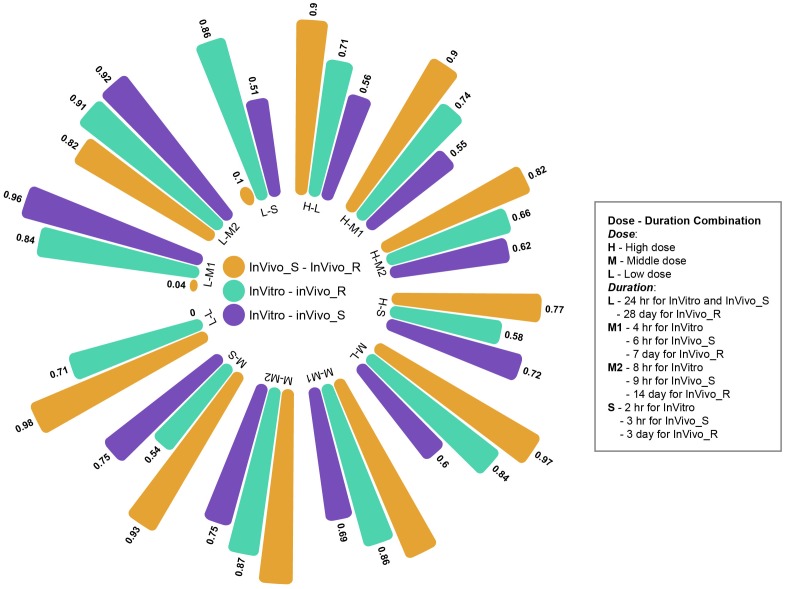
Circle bar plot for the concordance among three rat toxicogenomics systems at different time and dose combinations: the concordance between assay systems were conducted under a total of 12 time/dose combination by using Prank strategy.

The multiple time and dose combination design of TG-GATEs data sets provides a great opportunity to fully evaluate the pharmacokinetic and pharmacodynamic characteristics of chemical-induced toxicity and further facilitate early predictive biomarkers development for toxicity prediction and prevention. In the main part of this study, we comprehensively investigated the concordance among the three rat TGx assay systems at high dose and longest duration condition. Moreover, we expanded the comparisons to the different time and dose combinations. **Figure 5** shows the concordance among three assay systems at different time and dose conditions based on proposed PRank method. The circle bar plot represented the PRank scores. The similar trends of PRank scores changes (InVivo_S-InVivo_R > InVitro-InVivo_R > InVitro-InVivo_S) could be observed in high and medium dose with long and middle treatment duration. However, the low dose and short treatment durations were not able to provide enough discrimination power to assess the concordance among three TGx testing systems. Furthermore, the N-way ANOVA analysis were used to estimate the resource of variances contributing to the concordance among the TGx testing assays by using MATLAB function *anovan.m*. It was indicated that dose was more dominated influential factor of the concordance between testing systems than treatment duration (see **Supplementary Table S4**).

## Discussion

Animal models are indispensable in drug development and risk assessment, although extrapolation from animal models to human responses remains a challenge (Shanks et al., 2009). A key focus of research into animal models is how they could better recapitulate the human toxicological and physiological environment and provide a more reliable and robust prediction of human toxicity. Cell-based *in vitro* assays and *in silico* approaches have been proposed that could refine, reduce or even replace animal models (Hamburg, 2011; Goodman et al., 2015). In support of this, it is key to gain a better understanding on the similarities and differences between data generated in cell-based assay (*in vitro*) systems and animal (*in vivo*) models. Previously, we assessed similarities in TGx data between rat and human primary hepatocyte cultures and rat liver after 28 days of repeated dosing for a number of drugs and chemicals (Liu Z. et al., 2017). Here, we carried out a comparative analysis among three frequently-used rat TGx assay systems (InVitro, InVivo_S, and InVivo_R) using our previously described Pair Ranking (PRank) methodology.

The data indicated that there was a high concordance between the two *in vivo* assay systems (24 h and 28 days), indicating a potential to use a short-term *in vivo* assay for some endpoints saving time and money. Furthermore, the *in vitro* TGx data set had a relatively high similarity to the standard 28-day *in vivo* repeated dose experiment data, suggesting a good correlation of *in vitro* with longer term treatment *in vivo*. However, there was a poor concordance between *in vitro* and the *in vivo* single dose (24 h) treatment. This observation is at first surprising but one explanation could be that extraction of hepatocytes into cell culture followed by 24 h of treatment represents a level of chemical/environmental stress more equivalent to 28 days of *in vivo* treatment compared with 24 h (single dose) *in vivo* where the liver may only just be responding to a new chemical stress. Specifically, gene activities associated with the survival cells of the hepatocytes reflect a level of the adaptation that resemble to these in the 28-day repeated dosing conditions.

All three TGx assay systems could distinguish compounds by therapeutic category. Among the 12 investigated therapeutic categories, the ***C10****-Lipid modifying agents* with highest stability ratios in all the three assay systems, indicating the high discrimination power. It is very interesting that, when the analyses were focused on specific pathways, several pathways such as lipid metabolism-related pathways were consistently over-represented in all three assay systems, the finding is consistent with the therapeutic categories, suggesting that similarity between the systems is to some extent dependent on different biological process and compounds under different therapeutic categories.

It is worthwhile to consider some additional studies to further our knowledge and confirm the findings from this study. Firstly, the current comparisons among the three TGx assay systems were based on the perturbation of gene expression within each of these assay systems. Although this could be the case, there is no certainty that these conclusions are applicable to other assay systems where there may be differences in intrinsic properties such as species or tissue type and extrinsic properties such as time of exposure and culture conditions. Therefore, we proposed more retrospective analyses of preclinical TGx data sets should be undertaken to provide a boarder and more comprehensive picture of how animal models and cell-based *in vitro* assay systems can be translated to predict human responses. Secondly, in this study we employed TG-GATEs datasets, currently the largest dataset in the TGx research arena. Despite this, there are still many classes of chemicals and drugs missing. Therefore, more comprehensive and larger scale TGx datasets could yield more robust conclusion. Thirdly, in the current study, transcriptomic profiles (gene expression) data were used. With the advance of technology, other data such as microRNAs profiles should be investigated since these may be considered more conserved in different species and organ systems (Mack, 2007). Finally, in the current study we focused on the top 400 differentially expressed genes (DEGs) to reveal the relationship between testing systems. In our previous study, we have discussed the influence of the number of selected genes to the similarity measure and concluded that the selected 400 genes could generate the stable similarity ranking list in each assay system (Liu Z. et al., 2017). With that said, other methods and/or different lengths of DEGs applied should be consider to enhance the comprehensiveness of the investigation.

Toxicogenomics has been used widely to supplement risk assessment data, to elucidate underlying mechanisms of toxicology and to support predictive toxicology. One of the contentious questions in the toxicology field is whether animal models can provide sufficient predictive power for human toxicity. In this study, we investigated concordance among TGx data from three rat assay systems using a Pairwise Ranking strategy. The data generated provide an insight into the utility of these assay systems for drug safety evaluation and risk assessment.

## Author Contributions

Conceived and designed the experiments: ZL and WT. Analyzed the data and first version of the manuscript: ZL. Contributed reagents/materials/analysis tools: ZL and BD. Wrote the manuscript: ZL, RR, BD, and WT. All authors read and approved the final manuscript.

## Disclaimer

The views presented in this article do not necessarily reflect current or future opinion or policy of the U.S. Food and Drug Administration. Any mention of commercial products is for clarification and not intended as endorsement.

## Conflict of Interest Statement

RR is co-founder and co-director of ApconiX, an integrated toxicology and ion channel company that provides expert advice on non-clinical aspects of drug discovery and drug development to academia, industry and not-for-profit organizations. The other authors declare that the research was conducted in the absence of any commercial or financial relationships that could be construed as a potential conflict of interest.

## Footnotes

1 http://toxico.nibiohn.go.jp/english/

2 http://brainarray.mbni.med.umich.edu/Brainarray/Database/CustomCDF/CDF_download.asp

3 ftp://ftp.ncbi.nih.gov/pub/HomoloGene/

## Supplementary Material

FIGURE S1 Concordance of drug pairs between chemical space and assay systems: significant drug pairs in each assay system (Dice’s coefficient > 0.2) were used to investigate the concordance with chemical space by using Pearson’s correlation coefficients.

FIGURE S2 Twelve therapeutic categories with at least five compounds based on the second-level of Anatomical Therapeutic Chemical (ATC) Classification System system.

TABLE S1 Information of 120 common drugs among three rat toxicogenomics systems.

TABLE S2 106 toxicity pathways related genes sets with at least 200 genes from Comparative Toxicogenomics Database (CTD) database.

TABLE S3 Pairwise Dice’s coefficients in different time/dose/assay systems.

TABLE S4 Impact of time and dose for correction between assay systems base on N-way ANOVA analysis.
